# Efficacy and Tolerability of a Chemically Characterized *Scutellaria lateriflora* L. Extract-Based Food Supplement for Sleep Management: A Single-Center, Controlled, Randomized, Crossover, Double-Blind Clinical Trial

**DOI:** 10.3390/nu17091491

**Published:** 2025-04-28

**Authors:** Alessandro Di Minno, Maria Vittoria Morone, Daniele Giuseppe Buccato, Lorenza Francesca De Lellis, Hammad Ullah, Roberto Piccinocchi, Marcello Cordara, Danaé S. Larsen, Antonietta Di Guglielmo, Alessandra Baldi, Gaetano Piccinocchi, Xiang Xiao, Roberto Sacchi, Maria Daglia

**Affiliations:** 1 Department of Pharmacy, University of Napoli Federico II, Via D. Montesano 49, 80131 Naples, Italy; alessandro.diminno@unina.it (A.D.M.); lo.delellis2@gmail.com (L.F.D.L.); a.diguglielmo19@gmail.com (A.D.G.); alessandra.baldi.alimenti@gmail.com (A.B.); maria.daglia@unina.it (M.D.); 2 CEINGE-Biotecnologie Avanzate, Via Gaetano Salvatore 486, 80145 Naples, Italy; 3 Section of Microbiology and Clinical Microbiology, Department of Experimental Medicine, University of Campania “L. Vanvitelli”, 80138 Naples, Italy; mariavittoria.morone@unicampania.it; 4 School of Pharmacy, University of Management and Technology, Lahore 54000, Pakistan; 5 Level 1 Medical Director Anaesthesia and Resuscitation A. U. O. Luigi Vanvitelli, Via Santa Maria di Costantinopoli, 80138 Naples, Italy; roberto.piccinocchi@policliniconapoli.it; 6 School of Medicine, University of Milano-Bicocca, 20126 Milan, Italy; m.cordara@campus.unimib.it; 7 School of Chemical Sciences, The University of Auckland, Auckland 1010, New Zealand; d.larsen@auckland.ac.nz; 8 Comegen S.C.S., Società Cooperativa Sociale di Medici di Medicina Generale, Viale Maria Bakunin 41, 80125 Naples, Italy; gpiccino@tin.it; 9 School of Food and Biological Engineering, Jiangsu University, Zhenjiang 212013, China; xiaoxiang1@aliyun.com; 10 Applied Statistic Unit, Department of Earth and Environmental Sciences, University of Pavia, Viale Taramelli 24, 27100 Pavia, Italy; roberto.sacchi@unipv.it; 11 International Research Center for Food Nutrition and Safety, Jiangsu University, Zhenjiang 212013, China

**Keywords:** primary insomnia, sleep-wake cycle, *Scutellaria lateriflora* L., sleep quality, sleep efficiency, randomized clinical trial

## Abstract

**Background/Objectives**: Primary insomnia is characterized by persistent sleeplessness that is not caused by medical, psychological, or environmental factors. It is defined by difficulty initiating or maintaining sleep for at least one month, leading to significant distress or impairment in daily functioning. This randomized, crossover, double-blind, placebo-controlled clinical trial aimed to assess the efficacy and tolerability of a *Scutellaria lateriflora* L. extract-based food supplement in subjects with mild to moderate primary insomnia. **Methods**: A total of 66 participants, aged 18–70 years, were randomly allocated into two groups (1:1 allocation ratio) and received either the food supplement (400 mg/day) or a placebo for 56 days, separated by a 28-day washout period. The clinical effectiveness of the food supplement was assessed using the Pittsburgh Sleep Quality Index (PSQI) as the primary outcome measure. Secondary outcomes included sleep-wake cycle parameters (sleep onset latency, sleep efficiency, and total sleep time) and a Visual Analog Scale (VAS). **Results**: A significant improvement in sleep-wake balance following supplementation with *S. lateriflora*, as reflected by enhanced scores in both primary and secondary outcomes, was observed. Furthermore, none of the participants reported adverse effects from the food supplement. **Conclusions**: Overall, these findings suggest that a *S. lateriflora* extract-based food supplement is a safe and effective strategy for restoring the sleep-wake cycle and improving quality of life in individuals with primary insomnia.

## 1. Introduction

Insomnia, the most prevalent sleep disorder, is defined by consistent difficulties in falling asleep, waking up from non-restorative sleep, and experiencing daytime impairment. It is a critical public health concern because of its prevalence and the challenges associated with its management. Growing evidence suggests that various medical and mental health conditions are significantly linked to insomnia. Diagnosing and treating insomnia requires a comprehensive sleep history to identify the risk factors, as well as maladaptive behaviors that interfere with sleep [1]. According to the *International Classification of Sleep Disorders*, *Third Edition*, a sleep disorder is defined as “*a persistent difficulty with sleep initiation*, *duration*, *consolidation*, *or quality that occurs despite adequate opportunity and conditions for sleep*, *resulting in some form of daytime impairment*” [2]. Insomnia manifests in different ways, including sleep-onset insomnia, which involves trouble falling asleep, and sleep-maintenance insomnia, characterized by frequent or prolonged nighttime awakenings. Additionally, late-onset insomnia involves early morning awakenings, accompanied by an inability to resume sleep [3].

Epidemiological studies indicate that 30–35% of the global population experiences sleep deprivation, with 9–11% suffering from chronic sleep disorders [4]. Individuals with sleep disturbances often face significant psychological, social, and emotional disruptions. Research has established a direct link between sleep disorders and an increased risk of cardiovascular disease, hypertension, diabetes mellitus, and mental health conditions [5]. Stress is closely associated with sleep difficulties, as disruptions in the sleep-wake cycle significantly affect cortisol rhythms and other circulatory hormones, such as catecholamines. These hormonal imbalances may elevate the risk of insomnia-related conditions, including diabetes, obesity, and cognitive impairment [6]. Although insomnia is expected to contribute to higher mortality rates, studies on its direct relationship with mortality remain inconsistent. However, sleep deprivation has been strongly linked to memory loss [7], dementia [8], depression [9], and cardiovascular disease [10].

Despite the prevalence of sleep disorders, nearly half of affected individuals have never taken remedies to address their condition, and most have not discussed their concerns with their physicians. Among those who sought medical advice, approximately half received a prescription for sleep aids [11]. Common pharmacological treatments, including benzodiazepines and non-benzodiazepine hypnotics, have been associated with adverse effects such as depression, withdrawal-related insomnia, increased mortality, and daytime drowsiness [12]. Additionally, the long-term efficacy of these medications is rarely studied [13]. Due to these issues and the growing penchant for nonpharmacological treatments, it is essential to provide evidence-based alternatives that can improve sleep quality without the risks associated with medication [14].

The use of food supplements for managing sleep disorders is widely recognized, with both medical professionals and the general public considering them for mild to moderate sleep-related issues [15]. A study conducted in Quebec found that 18.5% of participants used natural sleep aids [16]. Traditional herbal remedies for insomnia include valerian, chamomile, and lavender extracts [17]. While research suggests that kava kava may help reduce anxiety and promote sleep [18], its use has been discontinued due to potential hepatotoxic effects [19].

The *Scutellaria* genus comprises over 350 species worldwide. *Scutellaria lateriflora* L. has a long history of usage by Native Americans for promoting blood circulation, reducing nervous tension, and treating neurological disorders. It is commonly used in North America in infusions or capsules to treat epilepsy, bacterial infection-related neurological damage, insomnia, anxiety, neuralgia, and tranquilizer and barbiturate withdrawal [20,21]. The aerial parts of this plant are rich in polyphenols, essential oils, diterpenoids, and amino acids, where baicalein, baicalin, chrysin, and wogonin are the primary components with mood-enhancing effects.

An in vitro study on a dried hydroethanolic *S. lateriflora* extract at non-cytotoxic concentrations ranging from 5 to 30 ng/mL demonstrated a significant suppression of cortisol release. This ranged from 58 to 91% in H295R cells, suggesting the potential of *S. lateriflora* as a food supplement ingredient to control cortisol-mediated stress response [22]. Other in vitro studies indicate that baicalein acts as a weak ligand for benzodiazepine receptors, suggesting mild sedative effects mediated by GABA-A receptor interactions. Wogonin exhibits anxiolytic effects through benzodiazepine receptor interaction, and other flavones bind to serotonin receptor 5-HT7. Additionally, an ethanolic extract of *S. lateriflora* inhibits glutamic acid decarboxylase and GABA transaminase, potentially contributing to its anxiolytic activity [23].

The rationale for investigating *S. lateriflora* as an ingredient in a sleep-promoting food supplement lies in its traditional uses. However, despite its promise, the current in vivo evidence surrounding its sleep-promoting properties remains inconclusive. This highlights the need for well-designed randomized clinical trials to establish its functional properties for promoting good sleep. This study is designed to focus on the chemical characterization of *S. lateriflora* extract, using RP-UHPLC-MS analysis, and to assess the effectiveness and tolerability of a *S. lateriflora* extract-based food supplement in improving sleep quality among participants with primary insomnia, using validated measures of sleep outcomes.

## 2. Materials and Methods

### 2.1. RP-UHPLC-MS Analysis of S. lateriflora Extract

The aerial parts of *S. lateriflora* were used to obtain a dried hydroethanolic extract (standardized to contain 10% baicalin) supplied by the EPO S.r.l. (Milan, Italy). Stock solutions of the hydroethanolic extract of *S. lateriflora* were made by weighing 500 mg of extract and diluting with a solution of 50:50 *v*/*v* acidified water (0.1% *v*/*v* formic acid) and methanol to 10 mg/mL. Then, 1 mL of stock solution was filtered prior to analysis (0.45 µm and 0.20 µm Minisart RC 4 membrane filters). A Thermo Ultimate RS 3000 paired online with a Q-Exactive hybrid quadrupole Orbitrap mass spectrometer (Thermo Fisher Scientific, Bremen, Germany) equipped with a heated electrospray ionization probe (HESI II) was used for analysis. A Kinetex^®^ EVOTM 150 mm × 2.1 mm, 2.6 µm (L × I.D., particle size, Phenomenex^®^, Bologna, Italy) column was employed at a flow rate of 0.4 mL/min during RP-UHPLC. The mobile phases were (A) 0.1% CH_3_COOH in H_2_O and (B) ACN plus 0.1% CH_3_COOH. The analysis was conducted using the following gradient protocol: 0–10.0 min, 2–35% B; 10–12 min, 35–70% B; 12–13 min, 70–98% B; hold for 2 min; and revert to the initial conditions after 0.1 min. Then, 5 µL of the extracts were injected into the column oven (40 °C). An HRMS analysis was executed with Full MS (*m*/*z* 100–850) and data-dependent acquisition (dd-MS2 top; N = 5). The resolution selected was 70,000 and 15,000 FWHM at *m*/*z* 200. Stepped normalized collision energy (NCE) was used with values of 15, 25, and 30. The negative ion mode (ESI-) was employed. Source parameters were as follows: sheath gas pressure, 50 arbitrary units; auxiliary gas flow, 13 arbitrary units; spray voltage, −2.50 kV; capillary temperature, 260 °C; auxiliary gas heater temperature, 300 °C; and S-lens RF value, 30 arbitrary units. Metabolite annotation was undertaken using Compound Discoverer (Thermo Scientific, V3.3, Waltham, MA, USA) and compared to in silico natural product libraries, accurate mass, and the existing literature as reported [24].

### 2.2. Food Supplement and Placebo

The food supplement ‘BlueCALM^®^’, which is registered with the Italian Health Ministry under notification number 164522, is a food supplement in capsule form, based on 400 mg *Scutellaria lateriflora* L. dried extract (standardized to contain 10% baicalin), along with other excipients such as hydroxypropyl methylcellulose (E464) as a coating agent and magnesium salts of fatty acids (E470b) as an anti-caking agent. The placebo was in the same capsule format, making it indistinguishable in color, flavor, shape, and weight from the food supplement, and was comprised of inert excipients, i.e., microcrystalline cellulose (E460(i)) as a filling agent, E464, and E470b. Both the food supplement and the placebo were manufactured by the FMC S.r.l. (Ferentino, Italy) to European specifications for contaminants and microbiological limits, and were provided free of charge by EPO S.r.l. (Milan, Italy).

### 2.3. Study Design

A single-center, controlled, randomized, crossover, double-blind clinical trial was conducted to evaluate the effectiveness of the food supplement. The study was double-blind for both the investigating physician and the enrolled subjects. The food supplement and placebo were indistinguishable in their packaging and dosage forms. Thus, the nature of the treatments remained undisclosed to all parties involved, including the sponsor, except in case of emergency. For emergency situations, investigators received sealed envelopes containing each participant’s treatment assignment. These envelopes could only be opened if necessary, such as upon a participant’s explicit request to be informed about their assigned treatment. Subjects were given oral and written information regarding the study before they provided their written consent. Protocol, letter of intent of volunteers, and synoptic documents on the study were approved by the Scientific Ethics Committee of Campania 1 (Resolution No. 658 of 29 March 2024) and carried out in accordance with the Helsinki Declaration of 1964 (as revised in 2000). This study is listed on the ISRCTN registry (www.isrctn.com) under the ID number ISRCTN12126820, https://doi.org/10.1186/ISRCTN12126820 (accessed on 26 February 2024).

Recruited participants were randomly allocated into two groups as follows: GROUP 1 (TREATMENT ORDER: treatment-control), initially received the treatment food supplement and, following a washout period, received the control (placebo). Similarly, GROUP 2 (TREATMENT ORDER: control-treatment order) received the placebo before a washout period and the food supplement after a washout period, following a crossover design. The eligibility of the subjects was evaluated through the application of the established inclusion and exclusion criteria during the trial screening. Additionally, an HIV test was conducted using a combined saliva rapid test (fourth-generation test) to detect anti-HIV antibodies and virus components, such as the p24 antigen. Moreover, the recruited subjects were asked not to change their dietary habits.

Clinical evaluations included the medical history of each subject, encompassing sleep history, psychiatric history, and sleep disturbances, including pain, work history, BMI, and waist circumference. A structured interview focused on sleep disturbances was administered, serving the purpose of classifying primary versus secondary insomnia. This interview was designed to collect comprehensive information and typically took 60–90 min. To gather detailed information, various questionnaires were utilized, including the Insomnia Severity Index (ISI) [25], which assessed the clinical relevance of insomnia, as well as the PSQI Questionnaire for evaluating sleep quality [26]. Furthermore, to eliminate subjects with undiagnosed depression or anxiety, the Patient Health Questionnaire-9 (PHQ-9) and the Generalized Anxiety Disorder 7 (GAD-7) were administered [27,28].

An integrated sleep diary was provided to each participant for the entire study duration (154 days). The study was then progressed through several treatment periods. At T0, the first treatment period started with the distribution of the sleep diary and placebo boxes, alongside the primary outcome evaluation via the PSQI questionnaire and secondary outcome evaluations. At T1, after 28 days, the same assessments were performed, including the collection of treatment data. By T2, the first treatment period was concluded after 56 days, followed by a washout period (28 days) where no treatment was administered, but daily logging of sleep diaries continued. At T3, the second treatment period started, during which the same evaluations were performed as in T0 and T1, but with the new treatment assignments. Finally, at T4 (28 days after the beginning of the second treatment) and T5 (56 days after the beginning of the second treatment), assessments continued through the designated follow-up evaluations to comprehensively analyze the primary and secondary outcomes, as established. The entire study spanned a total of seven months, comprising the various phases and evaluations highlighted above. In short, assessments were performed at various time intervals throughout the study: at baseline assessment (t0), and after 28 (t1) and 56 days (t2) for the first treatment period. Additionally, assessments were made at the baseline of the second treatment period (t3), after 28 days (t4), and after 56 days (t5) of the second treatment period, following a crossover design.

### 2.4. Participants and Recruiting

Sixty-six subjects, who reported that they had insomnia problems and met the inclusion and exclusion criteria, were recruited in an outpatient setting by the principal investigator. The subjects did not receive any financial compensation, and the food supplement was provided free of charge. The participants (aged 18–70 years, of either sex) were then randomized into two groups, with 33 subjects assigned to each. Inclusion criteria required that subjects had primary moderate insomnia persisting for at least one month, with Insomnia Severity Index (ISI) scores below 22, Generalized Anxiety Disorder (GAD-7) scores below 5, and Patient Health Questionnaire-9 (PHQ-9) scores below 5, suggesting that the subjects who were recruited did not suffer from any pathological anxiety or depression. Additionally, participants needed to be capable of understanding and signing the informed consent forms. Subjects were excluded if they were younger than 18 or older than 70 years, pregnant or breastfeeding, or had secondary insomnia or other sleep disorders, including obstructive sleep apnea, narcolepsy, night myoclonus, restless legs syndrome, phase advance or delay disorders, paradoxical insomnia, or parasomnias. Those with ISI scores exceeding 21 (severe insomnia), GAD-7 scores above 5, or PHQ-9 scores above 5 were also ineligible. Further exclusions applied to individuals diagnosed with psychiatric illnesses such as major depression, generalized anxiety disorder, post-traumatic stress disorder, panic attacks, bipolar disorder, dementia, or schizophrenia, as well as those suffering from chronic or acute pain conditions (e.g., arthritis, fibromyalgia, back pain, headaches), respiratory diseases (e.g., asthma), diabetes, heart diseases, hyperthyroidism, gastroesophageal reflux, epilepsy, Parkinson’s disease, Alzheimer’s disease, or kidney diseases. Individuals with substance abuse issues involving alcohol, amphetamines, caffeine, or theine were also excluded, as were those taking medications such as benzodiazepines, foods and food supplements commonly used for their relaxing properties (for example, valerian and chamomile), antidepressants, diuretics, or monoamine oxidase inhibitors within 14 days prior to recruitment. Additionally, those practicing relaxation techniques were excluded. Moreover, individuals who had received antibiotics within the past month or, depending on the treatment duration, within the last six months, were ineligible. Those with cognitive disorders that might affect questionnaire responses, known allergies to the ingredients in either the active or placebo treatment, or acquired immunodeficiency from HIV were also excluded from the clinical trial.

The subjects were recruited by COMEGEN Soc. Coop. Sociale, located at Viale Maria Bakunin, 41 (Parco S. Paolo), 80126—Naples (Italy). Participants received one capsule daily of BlueCALM^®^, a dietary supplement already registered and available on the Italian market, which consists of 400 mg of an aerial part of *S. lateriflora* dried hydroethanolic extract, along with a placebo in a different order, depending on their group assignment. Prior to enrollment, eligible subjects received an information sheet detailing the clinical study, objectives, and methodology; an informed consent form, completed and signed by both the participant and the investigator in duplicate, and an information and consent form regarding personal data processing. To ensure an adequate sample size by the study’s conclusion, an additional 15% of subjects were enrolled beyond the number determined by the power analysis, bringing the total to 66 participants.

Following enrolment, subjects were randomly assigned to one of the two groups. Randomization was generated using STATA 16 statistical software (Stata Statistical Software: Release 16. College Station, TX: StataCorp LLC). Participants were allocated into treatment groups through simple randomization (1:1 allocation ratio) to minimize selection bias and ensure a balanced distribution of prognostic characteristics. The randomization list was securely concealed to prevent tampering or premature disclosure. Sealed, opaque, stapled, and numbered envelopes were prepared by an individual uninvolved in the trial’s clinical execution. These envelopes were stored in a locked cabinet, ensuring the allocation sequence remained confidential until assignment. Investigators, unaware of the treatment assignments, dispensed treatments by opening the next envelope sequentially.

### 2.5. Outcomes of Study

The primary objective of this study was to assess the efficacy of the dietary supplement based on the extract of aerial parts of *S. lateriflora* in improving sleep-wake balance, following an intention-to-treat analysis. Specifically, the study assessed the effectiveness of a food supplement in maintaining proper sleep-wake balance, with a focus on sleep quality. Evaluation methods included the PSQI score, a validated questionnaire for self-assessment of sleep quality. The PSQI consisted of 9 questions, each scored from 0 to 3. A score of 0 designates no difficulty, whereas a score of 3 designates severe difficulty. Scores were categorized into seven components: subjective sleep quality, sleep latency, sleep duration, habitual sleep efficiency, sleep disturbances, use of sleep medications, and daytime dysfunction. The sum of all component scores resulted in a total PSQI score ranging from 0 to 21. A score of 0 signified no sleep difficulties, whereas a score higher than 21 indicated severe difficulties in all areas.

Secondary objectives of the study included evaluating sleep-wake cycle parameters, which were recorded using a sleep diary [29]. These parameters consisted of Total Time in Bed (TTL), which measured the time (in minutes) between when the subject goes to bed and when subject gets up, and Sleep Onset Latency (SOL), which refers to the time (in minutes) from when the subject turns off the lights or TV to when they fall asleep; Number of Awakenings (NR), which counts the number of nighttime awakenings; and Wakefulness After Sleep Onset (WASO), which quantifies the total wake time (in minutes) during nighttime awakenings. Additionally, Total Sleep Time (TST) was calculated, representing the time (in minutes) from when the subject falls asleep to when they wake up, minus any wakefulness during the night. Finally, the Sleep Efficiency Index (SEI) was determined using the formula: $SEI=\left(\frac{TST}{TTL}\right)\times 100$ [29]. Finally, the Visual Analogue Scale (VAS) was also recorded. The VAS is commonly used by healthcare providers to estimate pain intensity in adult patients and to evaluate pain relief following treatment. In sleep studies, it is often employed to measure the feeling of waking up refreshed [30].

### 2.6. Data Collection

Data collection was conducted using a structured Case Reporting Form (CRF) divided into two main sections. The first section captured personal data, subject history, any concomitant medication use, and treatment group, and it was completed at the time of enrollment. The second section documented the results of various questionnaires administered during the study. These included a structured sleep interview, the GAD-7 Questionnaire, the PHQ-9 Questionnaire, the ISI Questionnaire, and the PSQI Questionnaire, all of which were completed only during the screening visit, except for the PSQI Questionnaire and VAS Scale, which have been used at additional time points. In addition to these assessments, sleep parameters obtained from the integrated sleep diary were documented. Furthermore, any adverse effects were evaluated using a specific form modeled after the one employed by the Italian National Health Institute, Ministry of Health, for reporting suspected adverse reactions related to dietary supplements.

### 2.7. Safety and Tolerability

The ingredients contained within the food supplement were permitted by existing food regulations and considered safe. While the supplement was not expected to cause any adverse effects, participants were still closely monitored throughout the study. If any suspected adverse reactions occurred, these were documented through the VigiErbe online phytovigilance system (www.vigierbe.it) [31] in accordance with the provisions of the Istituto Superiore di Sanità. If Suspected Unexpected Serious Adverse Reactions (SUSARs) occurred during the clinical trial, these were to be submitted to the Ethics Committee, which had issued the approval for the trial.

### 2.8. Statistical Analysis

Sample size determination utilized a power analysis incorporating three power levels (1-β = 0.80, 0.95, and 0.99), an effect size (f = 0.15), and a significance level (α = 0.05). Power analysis, assuming the absence of carry-over effects, specifically focused on the interaction between measurement time and treatment to assess any variation in the treatment effect across different measurement points. This consideration influenced sample size calculations to ensure detection of a significant interaction. Thus, the final sample size was 66 participants, accounting for a 15% potential dropout rate.

Preliminary checks on outcome variables for normality by visual inspection indicated no substantial deviations. However, zero-inflation for the VAS was detected. Consequently, PSQI, Sleep Onset Latency, Sleep Effectiveness, and Total Sleep Time were modeled using a random intercept linear mixed model (LMM), whereas the VAS was modeled using a zero-inflated generalized linear mixed model (GLMM).

The LMMs incorporated the outcome variables as the dependent variable (each in a separate model), with measurement time points (t0, t3, and t5), treatment group (placebo vs. supplement), treatment order (placebo-supplement vs. supplement-placebo), and their three-way interaction as fixed effects. This interaction accounted for the effect of the treatment order on the differential effect of treatments over measurements (i.e., the two-way interaction measurement time × treatment group). In other words, the three-way interaction allowed us to detect and control possible crossover effects due to the experimental design. Finally, subject identity was included as a random effect to manage repeated measurements within subjects. The lme4 [32] package in R ver. 4.0.1 [33] was used for the analyses.

The GLMM incorporated the VAS, which showed a high proportion of zeros, as the response variable. The fixed and random components were the same as in the LMMs, while the zero-inflated component was modeled using a constant formula, assuming a uniform probability of excess zeros across observations. The GLMM was fitted using the glmmTMB package (version 1.1.11) in R. Unless specified differently, data are expressed as means and standard errors.

## 3. Results

### 3.1. Chemical Profile of S. lateriflora Extract

Chemical characterization of the commercial dried hydroalcoholic extract obtained from the aerial parts of *S. lateriflora* using RP-UHPLC coupled with a Q Exactive hybrid quadrupole-Orbitrap mass spectrometer was conducted. Through a comparison with the in silico MS/MS spectra, accurate mass, and molecular formula, 117 compounds were tentatively annotated in *S. lateriflora* extract, respectively, with confidence MSI lvl.2 [34], as reported in the Supplementary Materials (Table S1). Detailed characterization of metabolites showed the presence of phenolic acids, flavonols, flavones, flavanones, phenylethanoids, and *C*-glycosides.

### 3.2. Efficacy and Tolerability of Food Supplement

**Figure 1:**
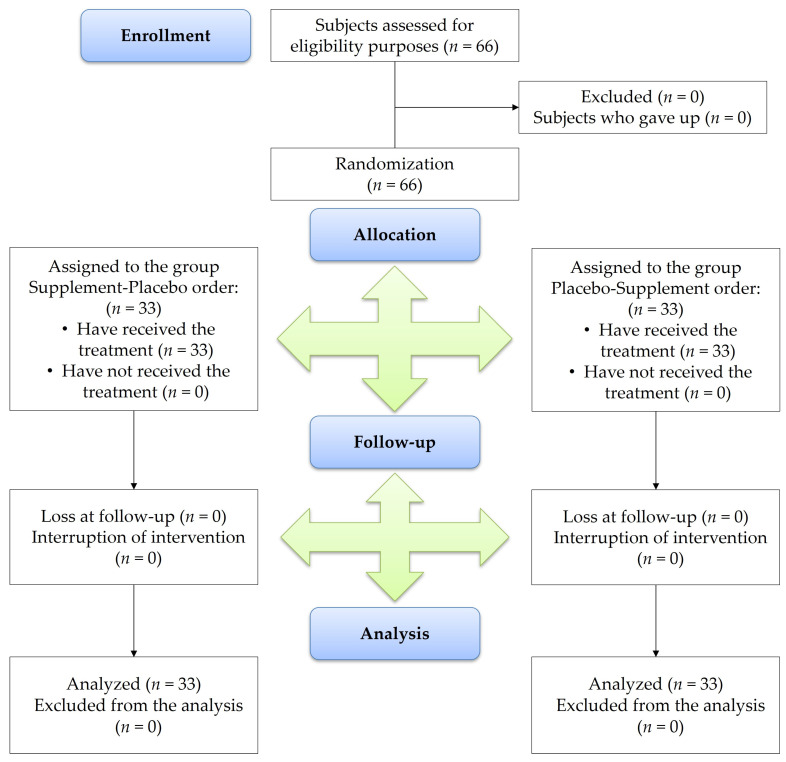
CONSORT flow diagram.

Figure 1 illustrates the study flow diagram, developed in accordance with the CONSORT PRO reporting standards [35]. Table 1 details the demographic and clinical characteristics of participants at baseline (T0). The study sample comprised 33 individuals per experimental group, with a total of 29 women and 37 men. The mean age (±SD) of participants was 44 ± 15 years (men: 43 ± 15 years for men and 45 ± 16 years for women). Table 2 presents the descriptive statistics comparing the placebo and food supplement groups at T0, T1, and T2 across the selected variables. The PSQI scores increased over time in the placebo group, indicating a progressive deterioration in sleep quality, whereas a consistent reduction in PSQI scores from T0 to T2 was observed in the food supplement group. Similarly, other sleep-related parameters, including sleep onset latency, sleep effectiveness, total sleep time, and VAS scores, demonstrated improvements in the food supplement group, supporting its potential efficacy in alleviating symptoms of mild to moderate sleep disturbances.

**Figure 2:**
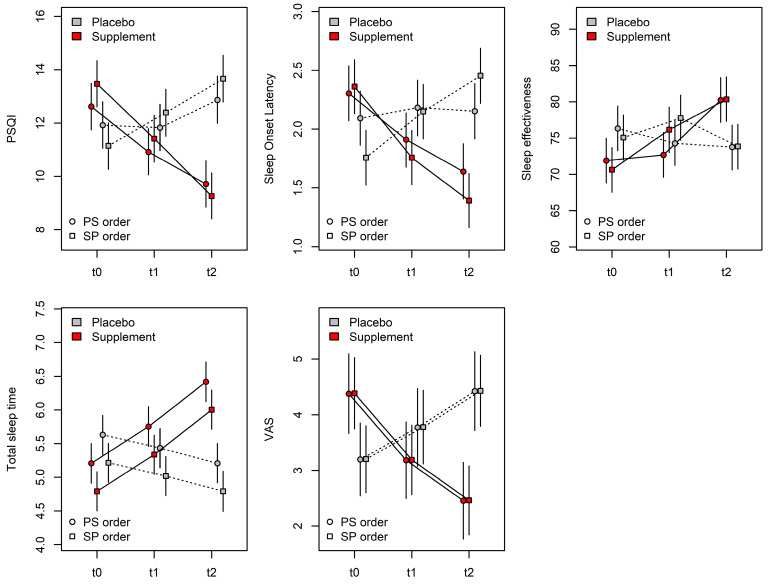
Comparison between the supplement and placebo treatments in the five related variables measured at times t0, t1, and t2, as predicted by the LMM (PSQI, Sleep Onset Latency, Sleep Effectiveness, and Total Sleep Time) and GLMM (VAS) models (means and 95% confidence intervals).

The LMM model for the PSQI questionnaire score identified a significant effect of measurement (*p* < 0.001), treatment (*p* < 0.001), their interaction (*p* < 0.001), the three-way interaction measure × treatment × order (*p* < 0.001), and the subjects’ age (*p* < 0.001). No significant effects were found for the gender of the enrolled subjects. This indicates that the PSQI questionnaire score varies between successive measurements differently for subjects first treated with placebo and those first treated with the food supplement. These differences are influenced by the order in which the treatments were administered. In particular, the PSQI score in the group treated with the supplement significantly decreased between t0 and t1 (β = 1.88 ± 0.31, t_320_ = 6.129, *p* < 0.001, Figure 2) and between t1 and t2 (β = 1.68 ± 0.31, t_320_ = 5.487, *p* = 0.0038, Figure 2), both in the placebo-supplement order and in the supplement-placebo order. In this regard, no significant differences in PSQI values were observed between treatment orders (placebo-treatment vs. supplement-placebo) within the same measurement (β < 0.86 ± 0.63, t_158_ < 1.364, *p* > 0.17, Figure 2). Administration of the placebo resulted in a non-significant increase in the PSQI score between t0 and t1 (β = 0.57 ± 0.31, t_320_ = 1.878, *p* = 0.061, Figure 2), but this was significant between t1 and t2 (β = 1.15 ± 0.31, t_320_ = 3.757, *p* < 0.001, Figure 2). Similarly, for placebo measurements, no significant differences were found between treatment orders within the same measurement (β < 0.80 ± 0.63, t_158_ < 1.268, *p* > 0.21, Figure 2). At measurement t1, this difference reversed, with a significantly lower score in participants taking the food supplement (β = 0.94 ± 0.31, t_320_ = 3.065, *p* = 0.0024, Figure 2), and the difference further increased at t2 (β = 3.77 ± 0.31, t_320_ = 12.308, *p* < 0.001, Figure 2). However, this trend was influenced by the treatment order: at t0, the difference between placebo and supplement was significant only with the order control-treatment (β = 2.33 ± 0.43, t_320_ = 5.383, *p* < 0.001, Figure 2) but not in the supplement-placebo order (β = 0.70 ± 0.43, t_320_ = 1.608 *p* = 0.11), Figure 2).

The LMM model for sleep onset latency (Table 3) identified significant effects for measurement (*p* < 0.001), treatment (*p* < 0.001), their interaction (*p* < 0.001), the three-way interaction measure × treatment × order (*p* < 0.001) and subjects’ age (*p* = 0.040). No significant effects were found for sex. This suggested that sleep onset latency varied between successive measurements differently for subjects treated with the placebo and those treated with the supplement, with these differences being influenced by the order of treatment administration. In particular, when treated with the food supplement, sleep onset latency was significantly reduced between t0 and t1 (β = 0.50 ± 0.09, t_320_ = 5.522, *p* < 0.001, Figure 2) and between t1 and t2 (β = 0.32 ± 0.09, t_320_ = 3.514, *p* < 0.001, Figure 2), both in the control-treatment and treatment-control order. In this regard, no significant differences were observed between the two orders (placebo-supplement vs. supplement-placebo) within the same measurement (β < 0.24 ± 0.17, t_198_ < 1.442, *p* > 0.15, Figure 2). Administration of the placebo resulted in a significant increase in sleep onset latency between t0 and t1 (β = 0.24 ± 0.09, t_320_ = 2.677, *p* = 0.0078, Figure 2), but no significant change between t1 and t2 (β = 0.13 ± 0.09, t_320_ = 1.506, *p* = 0.13, Figure 2). However, treatment order significantly affected the response to placebo, as these differences were not significant when the placebo was administered in a treatment-control sequence (β < 0.09 ± 0.09, t_320_ < 0.710, *p* > 0.15, Figure 2). Regardless of measurement, treatment, or administration order, the sleep onset latency significantly increased with age (β = 0.13 ± 0.06, t_63_ = 2.207, *p* = 0.031, Figure 2).

At measurement t0, the value of sleep onset latency was significantly higher in the supplement treatment compared to the placebo treatment (β = 0.41 ± 0.09, t_320_ = 4.518, *p* < 0.001, Figure 2). At t1, this difference reversed, with sleep onset latency being significantly lower in the supplement treatment (β = 0.33 ± 0.09, t_320_ = 3.681, *p* < 0.001, Figure 2), and the gap further increased at t2 (β = 0.79 ± 0.09, t_320_ = 8.700, *p* < 0.001, Figure 2). Once again, this trend was influenced by treatment order: at t0, the difference between placebo and supplement was significant only with the control-treatment order (β = 0.61 ± 0.13, t_320_ = 4.732, *p* < 0.001), whereas it was not significant in the treatment-control order (β = 0.21 ± 0.13, t_320_ = 1.656 *p* = 0.10, Figure 2).

The LMM model for sleep efficiency (Table 3) identified significant effects only for measurement (*p* < 0.001) and the measurement × treatment interaction (*p* < 0.001). No significant effects emerged for the three-way interaction, treatment order, age, or sex of the subjects. This suggested that sleep efficiency varies across successive measurements differently for subjects treated with the placebo and those treated with the supplement, without any influence from the treatment order. Specifically, sleep efficiency significantly increased with the supplement between t0 and t1 (β = 3.13 ± 1.46, t_323_ = 2.145, *p* = 0.033, Figure 2) and between t1 and t2 (β = 5.87 ± 1.46, t_323_ = 4.028, *p* < 0.001, Figure 2). In contrast, placebo administration had no significant effect on sleep efficiency, neither between t0 and t1 (β = 0.31 ± 1.46, t_323_ = 0.212, *p* = 0.83, Figure 2) nor between t1 and t2 (β = 2.20 ± 1.46, t_323_ = 1.512, *p* = 0.13, Figure 2). At t0, sleep efficiency was significantly higher in the placebo treatment compared to the supplement treatment (β = 4.41 ± 1.46, t_323_ = 3.029, *p* = 0.0026, Figure 2). However, at t1, no statistically significant difference was observed between treatments (β = 1.60 ± 1.46, t_323_ = 1.097, *p* = 0.27, Figure 2), while at t2, sleep efficiency was significantly higher in the supplement treatment (β = 6.48 ± 1.46, t_323_ = 4.444, *p* < 0.001, Figure 2).

The LMM model for total sleep time (Table 3) identified significant effects of measurement (*p* < 0.001), treatment (*p* < 0.001), their interaction (*p* < 0.001), treatment order (*p* = 0.027), and subjects’ age (*p* < 0.001). No significant effects were observed for the three-way interaction or gender. This suggested that the number of hours of sleep varied across successive measurements differently for subjects treated with the placebo and those treated with the supplement, without direct effects from the treatment order. In particular, the supplement led to a significant increase in sleep duration between t0 and t1 (β = 0.55 ± 0.12, t_325_ = 4.545, *p* < 0.001, Figure 2) and between t1 and t2 (β = 0.67 ± 0.12, t_325_ = 5.555, *p* < 0.001, Figure 2). Conversely, placebo administration had no significant effect on sleep duration either between t0 and t1 (β = 0.20 ± 0.12, t_325_ = 1.641, *p* = 0.10, Figure 2) or between t1 and t2 (β = 0.23 ± 0.12, t_325_ = 1.894, *p* = 0.060, Figure 2). At t0, subjects in the placebo group slept significantly longer than those in the supplement group (β = 0.42 ± 0.12, t_325_ = 3.535, *p* < 0.001, Figure 2). At t1, this pattern reversed, with subjects receiving the supplement sleeping significantly longer (β = 0.32 ± 0.12, t_325_ = 2.652, *p* = 0.0084, Figure 2), and this become even more pronounced at t2 (β = 1.21 ± 0.12, t_325_ = 10.101, *p* < 0.001, Figure 2). Irrespective of measurement and treatment, subjects who received the placebo before the supplement (placebo-supplement) slept significantly longer than those who received treatment in the opposite sequence (supplement-placebo order) (β = 0.41 ± 0.18, t_63_ = 2.289, *p* = 0.0015, Figure 2). Additionally, independent of measurement, treatment, and administration order, sleep duration significantly decreased with age (β = 0.30 ± 0.09, t_63_ = 3.311, *p* < 0.001, Figure 2).

The GLMM model for the VAS (Table 4) identified significant effects for measurement (*p* < 0.001), the treatment (*p* < 0.001), and their interaction (*p* < 0.001), while no significant effect was detected for the three-way interaction measure × treatment × order (*p* = 0.27). No significant effects were observed for gender, while a significant effect was detected for age (*p* < 0.001). This suggested that the VAS score varied across successive measurements differently among subjects treated with the placebo and those treated with the food supplement, and these differences were not influenced by the order of treatment administration.

Specifically, in the food supplement group, the VAS score significantly decreased between t0 and t1 (β = 1.19 ± 0.31, z = 3.897, *p* < 0.001, Figure 2) and also between t1 and t2 (β = 0.73 ± 0.31, z = 2.321, *p* = 0.020, Figure 2). Administration of the placebo resulted in a non-significant increase in the VAS score between measurements t0 and t1 (β = 0.57 ± 0.31, z = 1.884, *p* = 0.060, Figure 2) and a significant increase between t1 and t2 (β = 0.65 ± 0.30, z = 2.184, *p* = 0.029, Figure 2).

Consequently, at t0, the VAS score was significantly higher in the supplement treatment compared to the placebo (β = 1.18 ± 0.29, z = 4.036, *p* < 0.001, Figure 2). At t1, the difference between treatments was not significant (β = 0.59 ± 0.32, z = 1.851, *p* = 0.064, Figure 2), whereas at t2, the VAS score was significantly lower after supplement treatment compared to the placebo (β = 1.97 ± 0.30, z = 6.481, *p* < 0.001, Figure 2). Irrespective of measurement, treatment, or administration order, the VAS score significantly increased with age (β = 1.67 ± 0.23, z = 7.352, *p* < 0.001).

Lastly, over the 56 days of treatment, no subjects reported adverse effects, and the physicians assessed that the food supplement could be considered well-tolerated.

## 4. Discussion

In this study, a chemically characterized *S. lateriflora* extract-based food supplement was evaluated over three time intervals (i.e., t0, t1, and t2) for its efficacy in improving sleep quality and its tolerability in subjects with mild to moderate insomnia, in a single-center, placebo-controlled, randomized, crossover clinical trial.

The comprehensive profiling of *S. lateriflora* extract using RP-UHPLC-MS revealed a diverse range of phytochemicals, predominantly flavonoids (flavonols, e.g., quercetin and kaempferol derivatives, flavones, e.g., luteolin and apigenin derivatives, and flavanones, e.g., dihydrokaempferol and hesperetin derivatives) and phenolic acids (e.g., gallic acid, chlorogenic acid, and ferulic acid). These bioactive compounds have been documented to have potent antioxidant, anti-inflammatory, and health-promoting characteristics [36]. Moreover, the presence of *C*-glycosides, phenylethanoids, and other metabolites further highlights the chemical diversity of these phytochemicals in *S. lateriflora* extract.

Regarding the clinical trial, EFSA guidelines were followed in the planning of this study [37]. The treatment response was measured using PSQI questionnaire scores as the primary outcome, and VAS, time to fall asleep, hours of sleep per night, and sleep efficiency as secondary outcomes. The recruited participants were observed throughout the crossover and follow-up periods. Treatment with the food supplement resulted in improved sleep quality and sleep efficiency in recruited subjects. The carry-over effect was observed in the food supplement-treated subjects, indicating the persistent effect of the supplement for at least one month, even after discontinuation of the treatment. Moreover, the food supplement was found to be well-tolerated by the enrolled participants.

PSQI is a 19-item self-reporting questionnaire that covers the last seven days, used by academics and clinicians to assess several aspects of sleep, such as insomnia, sleep quality, and use of sleep aids. The component scores assess subjective sleep quality, sleep latency, duration, habitual sleep efficiency, sleep disruptions, medication use, and daytime dysfunction. The global score, from 0 to 21, is the total of the component scores ranging from 0 (no difficulty) to 3 (severe difficulty). A score of more than 5 indicates significant sleep disruption [26]. The food supplement significantly improved sleep quality as measured by the PSQI questionnaire compared to placebo, with a significant effect for measurement, treatment, measurement × treatment interaction, measurement × treatment × order interaction, and age of the participants (*p* < 0.001). However, reduction in PSQI score did not reach the threshold value of 5, below which sleep can be considered good.

In conclusion, as far as PSQI score and sleep onset latency data are concerned, the food supplement based on *S. lateriflora* extract improved sleep quality, as evidenced by a significant decrease in the PSQI score in the treated group compared to the placebo group. However, this reduction did not reach the threshold value of 5, below which sleep quality is considered good. The carry-over effect observed in the group that received the supplement first, followed by the placebo, suggested that the washout period between treatments (28 days) was insufficient. This also indicated a persistence of the supplement’s effects for at least one month after discontinuation.

Secondary parameters, including sleep onset latency, sleep efficiency, total sleep time, and VAS score, were also improved considerably in recruited subjects with the intake of the food supplement. Regarding sleep onset latency, sleep efficiency, and total sleep time, significant effects were observed for measurement, treatment, and measurement × treatment interaction, indicating a significant improvement over time with the food supplement but not with the placebo. A significant measurement × treatment × order interaction was also observed for sleep onset latency, which indicates a notable effect of food supplement on the time of sleep when it was consumed first in order, rather than after the placebo. Regarding sleep efficiency, significant effects were observed for measurement and measurement × treatment interaction but not for measurement × treatment × order interaction, which indicates a notable influence of food supplement on sleep efficiency, regardless of the order of intake. The VAS index identified significant effects both for measurement × treatment and measurement × treatment × order interactions, indicating considerable effects of food supplement as compared to placebo, regardless of the order of intake.

Brock et al. [38] reported significant mood-enhancing outcomes of *S. lateriflora* extract (350 mg per day) in healthy participants experiencing persistent stress, mood swings, irritability, and poor sleep quality in a randomized, double-blind, placebo-controlled, crossover study. However, differently from our results, sleep patterns did not differ significantly between *S. lateriflora* and the placebo. The authors justified their results by explaining that insomnia was a feature of anxiety, and only results from the anxious population would likely yield relevant results [39]. While conducting a pilot survey amongst herbal medicine practitioners, Brock et al. [40] documented that 53% of practitioners used to prescribe *S. lateriflora* with the expectation of improved sleep quality, i.e., sleeping longer, less nighttime waking, ability to initiate sleep, and feeling refreshed in the morning [41]. Given the absence of clinical studies supporting the traditional use of *S. lateriflora* for altered sleep-wake states, it is considered that the biological basis of the activity of extracts from the aerial parts of *S. lateriflora* on mood may, in part, share mechanisms with its effects on sleep, e.g., modulation of GABAergic receptors [41,42]. Baicalin, baicalein, and wogonin, derived from *Scutellaria baicalensis* Georgi, have been shown to bind to the GABA_A_-benzodiazepine receptor. Wogonin and baicalein bind the benzodiazepine site on GABA_A_ receptors more strongly than baicalin [43]. However, a study revealed that the anxiolytic and sedative properties of baicalein are linked with GABAergic non-benzodiazepine sites [44]. Ruan et al. [45] observed anxiolytic and sedative effects of baicalein in a mouse model of post-traumatic stress disorder via the serotonergic system and spinal delta-opioid receptors. Administration of baicalin on male Sprague–Dawley rats resulted in biphasic regulation of spontaneous sleep-wake cycle, as it decreased slow wave sleep throughout the light period and amplified the slow wave sleep and rapid eye movement sleep across the dark period, through the suppression of interleukin (IL-1) activity and increased GABA_A_ receptor activity [46]. Hui et al. [47] tested wogonin (monoflavonoid, 5,7-dihydroxy-8-methoxyflavone) as a substitute for benzodiazepines in anxiety management and found that wogonin can enhance GABA-activated current in rat dorsal root ganglion neurons and express recombinant rat GABA_A_ receptors in Xenopus laevis oocytes.

Some extensively studied herbs reported in the literature for their sleep-promoting effects include valerian (*Valeriana officinalis* L.) and chamomile (*Matricaria chamomilla* L.). Clinical trials showed improved sleep latency and quality with valerian supplementation [48,49]; however, meta-analysis reports showed mixed results due to variability in dosages and study designs [50,51,52]. Chamomile has uses as a mild sedative and anxiolytic herb and is thought to promote good sleep [53]. A meta-analysis of clinical trials found improvement of sleep with chamomile supplementation, particularly for staying asleep or number of awakenings after sleep, though no improvement was seen in the duration of sleep, sleep efficiency, and daytime functioning measures [54]. Considering the chemical composition of *S. lateriflora*, many phenolic acids, and flavonoids are commonly present in valerian and chamomile extracts (i.e., apigenin, luteolin, and quercetin derivatives, and derivatives of cinnamic and benzoic acids), and could, at least partially, be responsible for the effects on sleep along with the typical compounds of *S. lateriflora* listed above, which can be responsible for the other properties shown in the present study. In fact, *S. lateriflora* improved sleep quality, efficiency, and wakefulness, with a carry-over effect, which is not commonly seen in studies on valerian and chamomile; thus, it suggests *S. lateriflora* is superior in terms of effectiveness for use in mild to moderate insomnia. However, larger clinical trials are essential to confirm the efficacy of *S. lateriflora* over a longer time and throughout a diverse population. Melatonin, a hormone secreted by the pineal gland in response to darkness, is one of the most used therapies for primary insomnia, as it has been known to improve sleep quality and total sleep time with a decrease in sleep onset latency [55,56]. However, melatonin directly regulates the sleep-wake cycle, making it effective for circadian rhythm disorders, including shift work disorder or jet lag, while *S. lateriflora* does not target circadian rhythm but rather improves general sleep quality and efficiency; thus, it can be more effective in subjects without circadian rhythm disorder.

The present study possesses strengths and limitations. The major strength is its robustly designed clinical trial, which determines the efficacy and tolerability of a food supplement based on *S. lateriflora*. The use of crossover design allowed participants to serve as their own control, thus reducing the impact of individual variability such as genetics, baseline sleep patterns, and lifestyle, and ensuring that the observed effects are due to the intake of *S. lateriflora* rather than individual variability. The demonstrated improvement in tested parameters on sleep quality, efficiency, and wakefulness as compared to the placebo provides strong evidence for its potential use as a natural sleep aid. Unlike valerian and chamomile, where the effect fades quickly after discontinuation, the observed carry-over effects in the current study suggest a prolonged effect of the *S. lateriflora* extract-based food supplement on sleep regulation. The predominant limitation of the current study is the subjective nature of sleep quality assessment, which can be influenced by subject perceptions, expectations, and placebo effects. In addition, the observed carry-over effect suggests that a 28-day washout period may be insufficient, as it makes it challenging to fully separate the effects of the food supplement from the placebo across the second phase of the crossover study.

## 5. Conclusions

In summary, the results of this study provide valuable insights into the potential benefits of *S. lateriflora* supplementation for enhancing sleep quality in subjects with mild to moderate primary insomnia who did not suffer from any pathological anxiety or depression. It has been shown that the *S. lateriflora* extract-based food supplement can improve sleep-wake balance, as demonstrated by improvement in scores from validated questionnaires, which suggests it is safe and effective for use in primary insomnia disorders. However, larger-scale, multi-center randomized clinical trials are required in the future to support the encouraging results of the current study and to provide clinicians with accurate information for the inclusion of *S. lateriflora* in clinical practice. In particular, future research should include a more diverse sample to explore the impact of other factors, such as lifestyles and baseline sleep disturbance, on the efficacy of *S. lateriflora*. Future studies also need to include objective measures such as actigraphy and polysomnography, which will provide more robust data. It is also important to consider a longer washout period to eliminate residual effects of the food supplement.

## Figures and Tables

**Table 1:** Baseline demographic and clinical data of the study population.

Characteristics of Enrolled Subjects	Group Treatment-Control Order (*n* = 33)	Group Control-Treatment Order (*n* = 33)
Mean age (years)	44.2 ± 15.5	44.1 ± 15.0
Men	39.2 ± 13.9	46.8 ± 15.5
Women	50.3 ± 15.6	40.3 ± 14.2
Gender		
Men	18	19
Women	15	14
Body mass index		
Men	20.8 ± 2.1	21.3 ± 1.2
Women	22.4 ± 2.4	21.0 ± 2.6
Abdominal circumference (cm)		
Men	90.8 ± 3.1	89.5 ± 2.3
Women	76.2 ± 1.4	75.7 ± 1.8
Ethnicity: Caucasian	33	33

**Table 2:** Descriptive statistics (mean, standard deviation, and range of values) for the five response variables measured at times t0, t1, and t2 in the two experimental treatments.

	Placebo			Supplement		
	t0	t1	t2	t0	t1	t2
PSQI	11.5 ± 3.0	12.1 ± 2.7	13.3 ± 3.0	13 ± 2.9	11.2 ± 2.7	9.5 ± 3.5
	(6–18)	(8–19)	(9–20)	(9–19)	(6–17)	(5–19)
Sleep Onset Latency	1.9 ± 0.8	2.2 ± 0.6	2.3 ± 0.7	2.3 ± 0.6	1.8 ± 0.7	1.5 ± 0.8
	(0–3)	(1–3)	(1–3)	(1–3)	(1–3)	(0–3)
Sleep Effectiveness	75.7 ± 10	76 ± 10.5	73.8 ± 9.6	71.3 ± 10.1	74.4 ± 10.8	80.3 ± 8.6
	(50–88)	(50–100)	(57–100)	(50–100)	(50–100)	(57–100)
Total Sleep Time	5.4 ± 1.0	5.2 ± 0.9	5.0 ± 1.0	5.0 ± 1.0	5.5 ± 1	6.2 ± 1.2
	(2–7)	(2–8)	(2–7)	(1–7)	(2–7)	(2–8)
VAS	2.1 ± 2.5	2.1 ± 2.7	3.0 ± 3.1	2.8 ± 3.1	1.7 ± 2.4	1.7 ± 2.2
	(0–8)	(0–7)	(0–8)	(0–8)	(0–7)	(0–9)

**Table 3:** Results of the LMM models for the comparison between supplement and placebo treatments.

Variable	F	Df	*p*-Value
**PSQI**			
Measurement	9.473	2.320	**<0.001**
Treatment	36.26	1.320	**<0.001**
Order	0.251	1.62	0.62
Sex	0.003	1.62	0.96
Age	38.40	1.62	**<0.001**
Measurement × Treatment	74.53	2.320	**<0.001**
Measurement × Order	0.665	2.320	0.51
Order × Treatment	0.099	1.320	0.75
Measurement × Treatment × Order	5.572	2.320	**<0.001**
**Sleep Onset Latency**			
Measurement	5.944	2.320	**<0.001**
Treatment	20.614	1.320	**<0.001**
Order	0.370	1.62	0.55
Sex	2.845	1.62	0.10
Age	4.411	1.62	**0.040**
Measurement × Treatment	44.521	2.320	**<0.001**
Measurement × Order	0.905	2.320	0.41
Order × Treatment	0.756	1.320	0.39
Measurement × Treatment × Order	6.747	2.320	**<0.001**
**Sleep Efficiency**			
Measurement	5.959	2.320	**<0.001**
Treatment	0.034	1.320	0.85
Order	0.246	1.62	0.62
Sex	0.029	1.62	0.87
Age	1.829	1.62	0.18
Measurement × Treatment	15.10	2.320	**<0.001**
Measurement × Order	2.812	2.320	0.062
Order × Treatment	0.018	1.320	0.89
Measurement × Treatment × Order	2.038	2.320	0.13
**Total Sleep Time**			
Measurement	10.87	2.320	**<0.001**
Treatment	28.44	1.320	**<0.001**
Order	5.109	1.62	**0.027**
Sex	0.200	1.62	0.66
Age	10.53	1.62	**<0.001**
Measurement × Treatment	46.82	2.320	**<0.001**
Measurement × Order	1.158	2.320	0.32
Order × Treatment	0.048	1.320	0.83
Measurement × Treatment × Order	2.033	2.320	0.13

**Table 4:** Results of the GLMM model for the comparison between supplement and placebo treatments.

Variable	X^2^	Df	*P*
**VAS**			
Measurement	17.96	2	**<0.001**
Treatment	16.29	1	**<0.001**
Order	0.003	1	0.98
Sex	2.409	1	0.12
Age	54.05	1	**<0.001**
Measurement × Treatment	52.12	2	**<0.001**
Measurement × Order	5.145	2	0.076
Order × Treatment	0.236	1	0.63
Measurement × Treatment × Order	2.590	2	0.27

## Data Availability

The original contributions presented in this study are included in the article. Further enquiries can be directed to the corresponding authors.

## Supplementary Materials

The following supporting information can be downloaded at: https://www.mdpi.com/article/10.3390/nu17091491/s1, Table S1: Identified compounds in *S. lateriflora* extract according to molecular formula, *m/z*, and the retention time (RT).

## Abbreviations

The following abbreviations are used in this manuscript:

CRF	Case Reporting Form
GABA	Gamma-aminobutyric acid
GAD-7	Generalized Anxiety Disorder 7
ISI	Insomnia Severity Index
LMM	Linear Mixed Model
NR	Number of Awakenings
PHQ-9	Patient Health Questionnaire-9
PSQI	Pittsburgh Sleep Quality Index
SEI	Sleep Efficiency Index
SOL	Sleep Onset Latency
TST	Total Sleep Time
TTL	Total Time in Bed
VAS	Visual Analog Scale
WASO	Wakefulness After Sleep Onset
