# Supplementary material for: Efficacy and Tolerability of a Chemically Characterized Scutellaria lateriflora L. Extract-Based Food Supplement for Sleep Management: A Single-Center, Controlled, Randomized, Crossover, Double-Blind Clinical Trial
Source: Nutrients. 2025 Apr 28;17(9):1491. doi: 10.3390/nu17091491 (PMC12073644; doi:10.3390/nu17091491)
Supplement: Supplementary file 1 [file nutrients-17-01491-s001.zip › nutrients-3558070-supplementary.pdf]

*Article*

# Efficacy and Tolerability of a Chemically Characterized *Scutellaria lateriflora* L. Extract-Based Food Supplement for Sleep Management: A Single-Center, Controlled, Randomized, Crossover, Double-Blind Clinical Trial

**Alessandro Di Minno [1,2,†], Maria Vittoria Morone [3,†], Daniele Giuseppe Buccato [1,*], Lorenza Francesca De Lellis [1], Hammad Ullah [4,*], Roberto Piccinocchi [5], Marcello Cordara [6], Danaé S. Larsen [7], Antonietta Di Guglielmo [1], Alessandra Baldi [1], Gaetano Piccinocchi [8], Xiang Xiao [9], Roberto Sacchi [10] and Maria Daglia [1,11]**

**Table S1.** Identified compounds in *S. lateriflora* extract according to molecular formula, *m/z*, and the retention time (RT)

| Name | Formula | Δ [ppm] | *m/z* | RT [min] | Reference Ion |
|---|---|---|---|---|---|
| Gallic acid | C7 H6 O5 | -4,59 | 169,0135 | 1,588 | [M-H]-1 |
| Chlorogenic acid | C16 H18 O9 | 0,76 | 353,0881 | 4,76 | [M-H]-1 |
| Chlorogenic acid dimer | C32 H36 O18 | 2,06 | 707,1844 | 5,597 | [M-H]-1 |
| Chlorogenic acid isomer | C16 H18 O9 | 0,41 | 353,0880 | 5,609 | [M-H]-1 |
| Chlorogenic acid isomer | C16 H18 O9 | 0,6 | 353,0880 | 6,059 | [M-H]-1 |
| Kaempferol-3-O-hexo rhamnoside isomer | C27 H30 O15 | 0,96 | 593,1518 | 6,312 | [M-H]-1 |
| Carthamidin 7-O-glucoronide | C21 H20 O12 | 1,18 | 463,0887 | 6,676 | [M-H]-1 |
| Ferulic acid | C10 H10 O4 | -3,16 | 193,0500 | 6,733 | [M-H]-1 |
| Pentahydroxyflavanone | C21 H22 O11 | 0,64 | 449,1092 | 6,846 | [M-H]-1 |
| Schaftoside | C26 H28 O14 | 0,48 | 563,1409 | 6,865 | [M-H]-1 |
| Quercetin-dihexoside | C27 H30 O17 | 0,74 | 625,1415 | 6,965 | [M-H]-1 |
| Myricetin-hexoside | C21 H20 O13 | 0,12 | 479,0832 | 6,966 | [M-H]-1 |
| Luteolin 7,3'-diglucuronide | C27 H26 O18 | 0,87 | 637,1052 | 7,011 | [M-H]-1 |
| Quercetin-hexoside | C21 H20 O12 | 0,56 | 463,0885 | 7,129 | [M-H]-1 |
| 6-C-Glucopyranosyldihydrokaempferol | C21 H22 O11 | 0,95 | 449,1094 | 7,222 | [M-H]-1 |
| Taxifolin | C15 H12 O7 | 0,32 | 303,0512 | 7,272 | [M-H]-1 |
| Quercetin 3-glucuronide | C21 H18 O13 | 0,79 | 477,0678 | 7,353 | [M-H]-1 |
| Schaftoside isomer | C26 H28 O14 | 0,53 | 563,1409 | 7,39 | [M-H]-1 |
| Luteolin 7-glucuronide | C21 H18 O12 | 0,98 | 461,0730 | 7,532 | [M-H]-1 |
| Ellagic acid | C14 H6 O8 | 0,38 | 300,9991 | 7,592 | [M-H]-1 |
| Rutin | C27 H30 O16 | 0,21 | 609,1462 | 7,595 | [M-H]-1 |
| 6-C-beta-D-Hexosyl apigenin | C21 H20 O10 | -0,27 | 431,0983 | 7,619 | [M-H]-1 |
| Kaempferol-3-O-hexo rhamnoside | C27 H30 O15 | 0,5 | 593,1515 | 7,642 | [M-H]-1 |
| Verbascoside | C29 H36 O15 | 0,65 | 623,1986 | 7,669 | [M-H]-1 |
| Quercetin-3-O-glucopyranoside (isoquercitrin) | C21 H20 O12 | 0,82 | 463,0886 | 7,686 | [M-H]-1 |
| Persicogenin-hexoside | C23 H26 O11 | 0,66 | 477,1406 | 7,754 | [M-H]-1 |
| Ferulic acid isomer | C10 H10 O4 | -3,44 | 193,0500 | 7,762 | [M-H]-1 |
| Quercetin-hexoside isomer | C21 H20 O12 | 0,72 | 463,0885 | 7,789 | [M-H]-1 |

| | | | | | |
|---|---|---|---|---|---|
| Kaempferol 3-hexoside-7-glucuronide | C27 H28 O17 | 0,38 | 623,1256 | 7,849 | [M-H]-1 |
| Chrysin 8-C-β-d-hexoside | C21 H20 O9 | 0,29 | 415,1036 | 7,945 | [M-H]-1 |
| Ferulic acid isomer | C10 H10 O4 | -3,63 | 193,0499 | 7,951 | [M-H]-1 |
| Quercetin 4'-methyl ether 3-hexoside | C22 H22 O12 | 0 | 477,1039 | 8,092 | [M-H]-1 |
| Kaempferol-3-O-hexo rhamnoside isomer | C27 H30 O15 | 0,7 | 593,1516 | 8,25 | [M-H]-1 |
| Isoorientin 3'-O-glucuronide | C27 H28 O17 | 0,48 | 623,1257 | 8,337 | [M-H]-1 |
| Dihydrokaempferol | C15 H12 O6 | 0,46 | 287,0562 | 8,382 | [M-H]-1 |
| Hesperetin 7-O-hexoside | C22 H24 O11 | 1,35 | 463,1252 | 8,384 | [M-H]-1 |
| Scutellarein 7,4'-dimethyl ether 6-sophoroside | C29 H34 O16 | 0,43 | 637,1777 | 8,442 | [M-H]-1 |
| Luteolin 3',4'-diglucuronide | C27 H26 O18 | 0,37 | 318,0487 | 8,471 | [M-2H]-2 |
